# Identification of DNA methylation markers for early detection of CRC indicates a role for nervous system-related genes in CRC

**DOI:** 10.1186/s13148-021-01067-9

**Published:** 2021-04-15

**Authors:** Glenn Rademakers, Maartje Massen, Alexander Koch, Muriel X. Draht, Nikkie Buekers, Kim A. D. Wouters, Nathalie Vaes, Tim De Meyer, Beatriz Carvalho, Gerrit A. Meijer, James G. Herman, Kim M. Smits, Manon van Engeland, Veerle Melotte

**Affiliations:** 1 Department of Pathology, GROW – School for Oncology and Developmental Biology, Maastricht University Medical Center, P.O. Box 616, 6200 MD Maastricht, The Netherlands; 2 Department of Data Analysis and Mathematical Modelling, Ghent University, Ghent, Belgium; 3 Department of Pathology, Netherlands Cancer Institute, Amsterdam, The Netherlands; 4 The Hillman Cancer Center, University of Pittsburgh Cancer Institute, Pittsburgh, PA USA; 5 Department of Clinical Genetics, Erasmus University Medical Center, Rotterdam, The Netherlands

**Keywords:** Colorectal cancer, Diagnostic markers, In silico discovery, DNA methylation, Nervous system

## Abstract

**Purpose:**

Colonoscopy and the fecal immunochemical test (FIT) are currently the most widely used screening modalities for colorectal cancer (CRC), however, both with their own limitations. Here we aim to identify and validate stool-based DNA methylation markers for the early detection of CRC and investigate the biological pathways prone to DNA methylation.

**Methods:**

DNA methylation marker discovery was performed using The Cancer Genome Atlas (TCGA) colon adenocarcinoma data set consisting of normal and primary colon adenocarcinoma tissue. The performance of the five best candidate markers and a previously identified marker, *NDRG4*, was evaluated on tissues and whole stool samples of healthy subjects and CRC patients using quantitative MSP assays. The results were compared and combined with FIT data. Finally, pathway and gene ontology enrichment analyses were performed using ToppFun, GOrilla and clusterProfiler.

**Results:**

*GDNF, HAND2, SLC35F3, SNAP91* and *SORCS1* were ranked as the best performing markers. Gene combinations of all five markers, *NDRG4* and FIT were evaluated to establish the biomarker panel with the highest diagnostic potential, resulting in the identification of *GDNF/SNAP91/NDRG4/FIT* as the best performing marker panel. Pathway and gene ontology enrichment analyses revealed that genes associated with the nervous system were enriched in the set of best performing CRC-specific biomarkers.

**Conclusion:**

In silico discovery analysis using TCGA-derived data yielded a novel DNA-methylation-based assay for the early detection of CRC, potentially improving current screening modalities. Additionally, nervous system-related pathways were enriched in the identified genes, indicating an epigenetic regulation of neuronal genes in CRC.

**Supplementary Information:**

The online version contains supplementary material available at 10.1186/s13148-021-01067-9.

## Introduction

Colorectal cancer (CRC) is the third most common type of cancer and the second cause of cancer-related mortality worldwide [1]. Implementing screening programs for early CRC detection leads to substantial reductions in CRC incidence and mortality [2]. Colonoscopy and the fecal immunochemical test (FIT) are the most widely used modalities for CRC screening [3, 4], but both have limitations. The invasive nature of colonoscopy is associated with risks of bleeding, bowel perforation, low participation rates and high costs, while the sensitivity and specificity of FIT are suboptimal [5, 6]. To improve the current FIT, alterations in CRC-derived DNA found in bodily fluids (e.g., blood and feces) have been evaluated [7]. Three DNA methylation markers (*SEPTIN9, NDRG4* and *BMP3*) have been incorporated in FDA-approved screening tests for early CRC detection [8]. Two assays detect *SEPTIN9* methylation in blood (Epi proColon® 2.0 CE and Colovantage®) with a sensitivity of 58.6–81.0% and a specificity of 80.0–99.0% [9–18]. The multitarget stool DNA test Cologuard®, combining *KRAS* mutations, *BMP3* and *NDRG4* methylation and an immunochemical assay for human hemoglobin [19, 20], reported a sensitivity of 92.3% and specificity of 86.6% [19, 21].

The observation that only 0.8% of the reported DNA methylation markers have been translated into a commercial product illustrates the complexity of translating laboratory discoveries to clinical applications [8]. Reasons for this include flawed study designs, suboptimal marker identification and methodological issues [8]. To improve clinical translation, we previously recommended (1) to critically evaluate the genomic location of DNA methylation biomarkers and (2) to use publicly available (epi)genomics databases [8].

Here, we applied these recommendations to optimize the implementation of early CRC detection DNA methylation biomarkers using publicly available data from The Cancer Genome Atlas (TCGA) [22]. Five candidate DNA methylation biomarkers were identified that were further validated in CRC tissue and stool samples. Many of the identified candidate markers were involved in nervous system-related pathways, indicating a role for the nervous system in colorectal carcinogenesis.

## Methods

For more detailed information, see supplemental methods.

### Gene discovery analysis

The following TCGA data sets for colon adenocarcinoma were used: clinical patient data, level 3 Infinium 450 k DNA methylation data and level 3 Illumina HiSeq RNA-seq V2 gene expression data (upper quartile normalized RSEM expression estimates). Methylation and gene expression data were available for 12,263 genes.

**Fig. 1:**
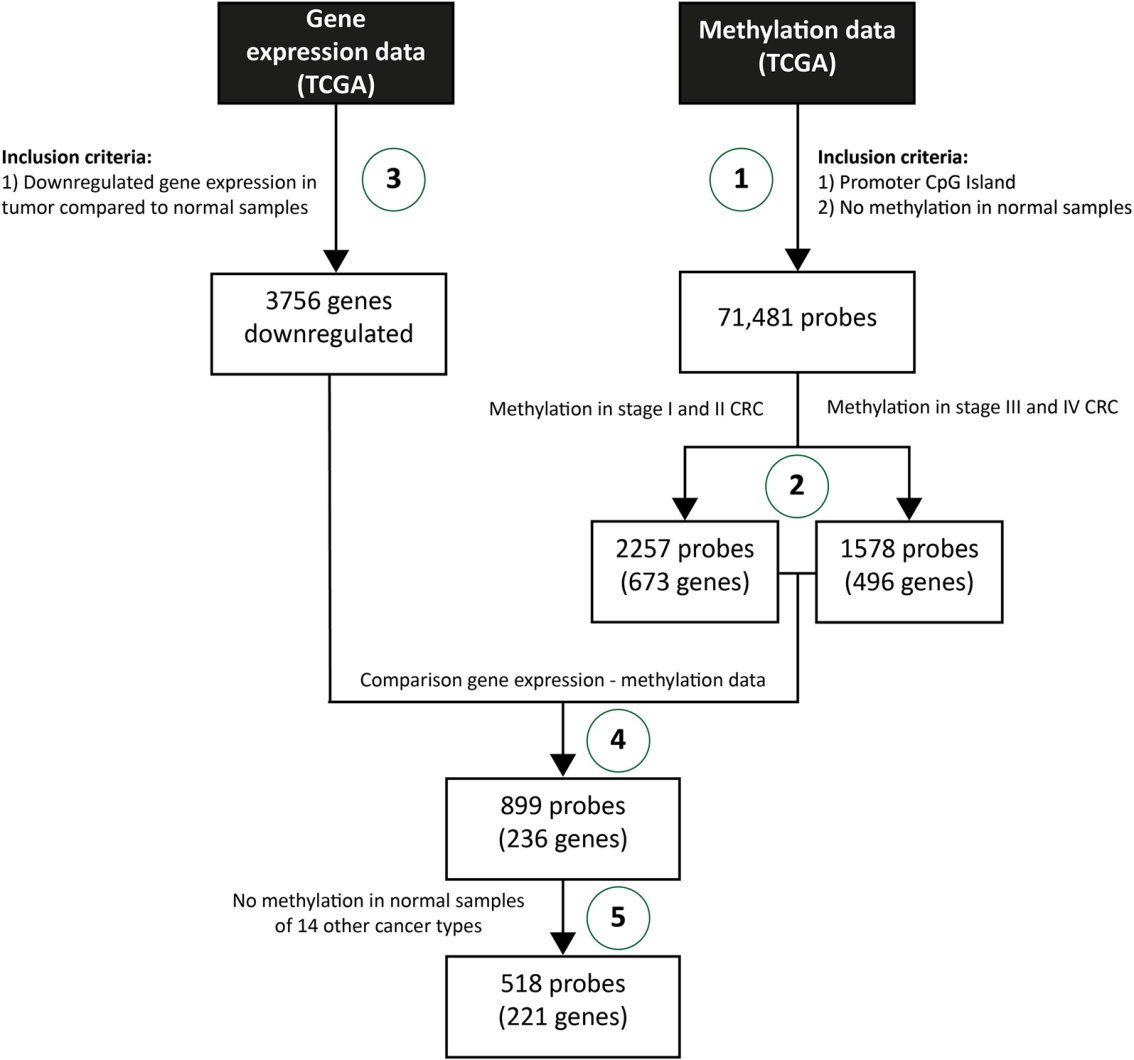
Pipeline to select candidate methylation markers using The Cancer Genome Atlas (TCGA) database. Marker discovery is based on a selection procedure using methylation data (right section) and gene expression data (left section). The DNA methylation analysis resulted in a list of Infinium 450 k probes that were: (1) located in promoter CpG islands, (2) unmethylated in normal colon tissue, and (3) hypermethylated in tumor samples over all four stages cancer development. This list was compared with a list of genes downregulated in tumor compared to normal samples, and we checked the methylation status of the remaining probes in normal samples from 14 different cancer types, resulting in a list of 221 genes. Finally, we designed and tested primers for the probes with the highest sensitivity and specificity based on the TCGA data, resulting in the top five potential early detection markers for CRC

TCGA methylation data were selected for the Infinium 450 k probes located in promoter CpG islands (i.e., the region from − 1000 bp to + 500 bp around the transcription start site) that were unmethylated in normal samples (median β over all normal samples < 0.20) (Fig. 1, “Introduction” section). Because a clinically relevant early detection marker needs to identify all tumor stages, we compared normal samples (*n* = 37) with stage I and II tumor samples (*n* = 146) and stage III and IV tumor samples (*n* = 116) using a one-sided Mann–Whitney test (*μ* = − 0.25). Resulting *P* values were corrected using false discovery rate (FDR) correction and only probes with an FDR < 0.05 and a difference in median *β* < − 0.25 were retained (Fig. 1, “Methods” section). Probes for which at least one other differentially methylated probe was located within 750 bp up or downstream were selected. Next, downregulated genes in the tumor samples were identified in the RNA-seq expression data (normal tissue: *n* = 41, primary tumor tissue: *n* = 285) with the one-sided Mann–Whitney test (*μ* = 1), using an FDR cutoff of 0.05 (Fig. 1, “Results” section). The results of both differential methylation analyses were combined with the results of the differential gene expression analysis yielding a list of 236 genes (Fig. 1, “Discussion” section) [8].

To ensure tumor-specificity of the methylation, we evaluated methylation of the selected probes in normal samples from fourteen TCGA cancer types, i.e., breast cancer, colon adenocarcinoma, lung squamous cell carcinoma, prostate adenocarcinoma, head and neck squamous cell carcinoma, lung adenocarcinoma, bladder urothelial carcinoma, kidney renal clear cell carcinoma, kidney renal papillary cell carcinoma, liver hepatocellular carcinoma, thyroid carcinoma, uterine corpus endometrial carcinoma, colorectal adenocarcinoma and esophageal carcinoma. For every probe and tissue type, the percentage of methylated (median *β* > 0.20) samples was calculated. Only probes for which the maximal fraction of methylated samples was lower than 25% in each tissue type were retained (Fig. 1, Sect. 5), revealing 221 genes. All analyses were performed using the R programming language (version 3.2.3).

### Tissue samples

Formalin-fixed, paraffin-embedded tumor tissue (CRC) and matching normal tissue was retrospectively collected from the tissue archives of the Pathology Department of the Maastricht University Medical Center (MUMC) for 34 patients diagnosed between 1995 and 2003 (Additional file 1: Table S1). This study was approved by the Medical Ethical Committee of the MUMC (MPTC 2015–12).

### Collection of FIT and stool samples

Stool samples from colonoscopy-negative controls (*n* = 50) were prospectively obtained from a workplace-based CRC screening study at MUMC. Stool samples from CRC 121 patients (*n* = 43) were prospectively collected in the noninvasive markers for CRC (NIM) study at MUMC. Both studies were approved by the Medical Ethical Committee of the MUMC (METC 04-088, METC 08-2-038) and written informed consent was obtained from all patients. Population characteristics are shown in Table 1.

**Table 1:** Clinicopathological features of the stool samples obtained from a hospital-based series

Patient demographics	Normal (*N* = 50 |*n* (%)|)	Carcinoma (*N* = 42 |*n* (%)|)
Sex		
Male	24 (48.0)	28 (66.7)
Female	26 (52.0)	14 (33.3)
Age (years)		
Median (± StDev)	55.5 (± 3.7)	70.5 (± 10.5)
Histological type		
Adenocarcinoma	–	31 (73.8)
Signet ring cell carcinoma	–	1 (2.4)
Mucinous adenocarcinoma	–	2 (4.8)
High-grade neuroendocrine carcinoma	–	1 (2.4)
Unknown		7 (16.6%)
Differentiation grade		
Poor	–	6 (14.3)
Moderate/well	–	25 (59.5)
Unknown	–	11 (26.2)
T-stage		
Stage 1	–	0 (0.0)
Stage 2	–	7 (16.7)
Stage 3	–	24 (57.1)
Stage 4	–	10 (23.8)
Unknown	–	1 (2.4)
Location		
Proximal	–	14 (33.3)
Distal	–	25 (59.5)
Unknown/other	–	3 (7.2)

Tissue retrieved retrospectively from the tissue archive of the department of Pathology of the Maastricht University Medical Center

^**±**^*P* < 0.000 compared to normal, one-way ANOVA was used

Participants collected one bowel movement in stool collection container (Exact Sciences) just before the bowel preparation for the colonoscopy. Directly after, stabilization buffer (Exact Sciences) was added. Simultaneously, FIT (OC-Sensor, Eiken Chemical, Tokyo, Japan) was performed, according to the manufacturer’s instructions. A quantitative concentration of 50 ng Hb/ml test buffer was set as threshold for a positive FIT. The FIT and stool samples were delivered to the laboratory within 72 h after collection, stored at − 20 °C and further processed as previously described [23].

### Methylation data and statistical analyses

Methylation frequencies of the selected genes were determined in carcinoma and matched normal tissues and compared with McNemar’s test. A receiver operating characteristic (ROC) curve analysis and area under the curve (AUC) were established to assess their diagnostic utility. For the AUC, a 95%-confidence interval was estimated using a nonparametric method. The qMSP cutoff value for each marker was determined based on the highest likelihood ratio (see Additional file 1: Supplementary methods). Promoter methylation was considered positive if the methylation value was higher than the predetermined cutoff. Next, the best performing marker panel was identified. The Pearson chi-square test was used to compare methylation frequencies in fecal DNA between CRC patients and healthy subjects. All statistical analyses were performed using IBM SPSS Statistics 23, R programming language (version 3.2.3) or Graphpad Prism (version 5.03).

### Gene ontology and pathway analyses

Gene ontology enrichment analyses using Gorilla [24] and clusterProfiler [25] were performed on the 221 differentially methylated and downregulated genes compared to the background gene set (12, 263 genes). Gene ontology analyses were performed on three different subsets of gene ontologies: cellular component, molecular function and biological process. The number of nervous system-related gene ontologies in the enriched sets was compared with the frequency of these ontologies in the complete set of ontologies (Gene Ontology Consortium http://geneontology.org/, link to the ontology file: http://purl.obolibrary.org/obo/go/go-basic.obo). To identify the number of neuronal-related gene ontologies, we used neuronal-related keywords (‘neuro,’ ‘neuron,’ ‘neuronal,’ ‘neural,’ ‘nervous,’ ‘axon,’ ‘dendritic,’ ‘synaptic,’ ‘synapse,’ ‘learning,’ ‘memory,’ ‘brain,’ ‘hippocampus’) and applied the Fisher’s Exact test to compare the frequencies of the neuronal-related ontologies (R version 3.2.3).

In addition, pathway analysis was performed using three major pathway databases (Reactome, KEGG and PantherDB) using the ToppFun application (http://toppgene.cchmc.org) [26]. Pathways identified with a cutoff value of *P* < 0.05 were considered for further analysis. As standard methods to correct *P* values for multiple testing tend to be conservative, these corrections were not applied.

## Results

### In silico identification of early CRC detection DNA methylation markers using publicly available TCGA data

A multistep in silico gene discovery analysis was used to identify novel candidate DNA methylation markers for early CRC detection (Fig. 1). The list of Infinium Human Methylation 450 K BeadChip probes (*n* = 485,577) was initially reduced to 71,481 probes based on location in promoter CpG islands and the absence of methylation in normal samples. Based on their methylation status, 2257 probes/673 genes in stage I/II and 1578 probes/496 genes in stage III/IV were identified. This list was combined with 3756 genes identified as downregulated in tumor compared to normal samples, resulting in 899 probes/236 genes. Finally, to select tumor specific probes, all 899 probes were investigated in normal samples of fourteen other cancer types, resulting in 518 probes/221 genes (Fig. 1). The top twenty genes with the highest sensitivity and 100% specificity were selected for further investigation.

### Methylation frequencies of the best performing genes in CRC tissue

After performing MSPs on 34 matched CRC and normal colon tissue samples, we obtained MSP data for ten genes (dropouts due to unsuccessful and suboptimal MSP primer design). We identified *GDNF*, *HAND2*, *SLC35F3*, *SNAP91* and *SORCS1* as the five best performing tissue candidates and selected these markers for further qMSP analysis. Methylation frequencies for these genes differed between CRC and normal tissue (*P* < 0.0001 for all genes) with high methylation in CRC; *SORCS1* (91.2%), *SLC35F3* (88.2%), *SNAP91* (85.3%), *GDNF* (76.5%) and *HAND2* (73.5%) (Additional file 2: Figure S1). In the matched normal tissue samples, methylation was lower; *HAND2* (8.8%), *SNAP91* and SORCS1 (both 5.9%), and *GDNF* and *SLC35F3* (both 2.9%) %) (Additional file 2: Figure S1). This in situ validation of in silico identified DNA methylation markers shows the potential of public data for biomarker discovery.

### Sensitivity and specificity for the best performing genes in fecal DNA of CRC patients

**Fig. 2:**
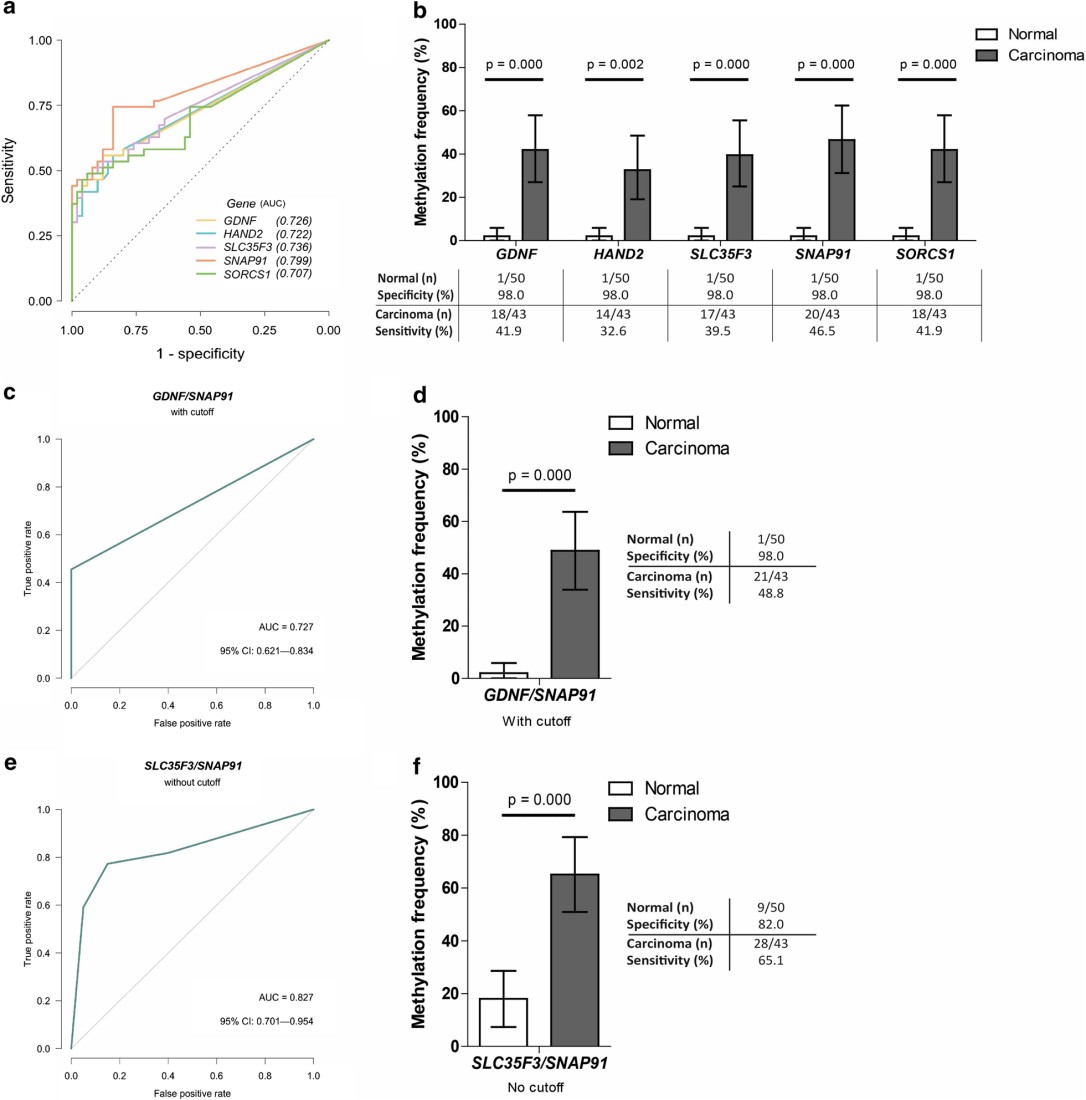
Early detection methylation marker validation using fecal DNA from healthy controls and CRC patients. **a** Receiver operating characteristic (ROC) curve for marker validation of *GDNF, HAND2, SLC35F3, SNAP91 and SORCS1* on fecal DNA to determine optimal sensitivity/specificity. The jagged lines indicate the different ROC curves for each independent marker. The dashed line represents the line of no discrimination between good and bad classification. **b** Methylation frequency (%) of single markers in fecal DNA of healthy controls (white bar) and CRC patients (black bar). The exact number of methylated samples is indicated in the table below for both groups (methylated samples/total number samples). For the healthy control group, the specificity is given; for carcinomas the sensitivity was determined. Pearson’s chi-square test was used to calculate *P* values. **c** ROC curve for the best performing marker panel (*GDNF*/*SNAP91*) based on the cutoffs for *GDNF* and *SNAP91*. The jagged (green) line indicates the ROC curve for this specific panel. The gray line represents the line of no discrimination between good and bad classification. **d** Methylation frequency of the *GDNF*/*SNAP91* marker panel in fecal DNA of healthy controls (white bar) and carcinomas (dark gray bar). Fisher’s exact test was used to calculate *P* values. **e** ROC curve for the best performing marker panel (*SLC35F3*/*SNAP91*) without cutoff. **f** Methylation frequency of the *SLC35F3*/*SNAP91* marker panel in fecal DNA of healthy controls (white bar) and carcinomas (dark gray bar). Fisher’s exact test was used to calculate *P* values

The diagnostic performance of the five identified DNA methylation markers in fecal DNA was assessed in stool samples from 50 healthy subjects and 43 CRC patients. The AUCs for *GDNF*, *HAND2*, *SLC35F3*, *SNAP91* and *SORCS1* in stool were 0.726 [95%-CI 0.619–0.834], 0.722 [95%-CI 0.615–0.829], 0.736 [95%-CI 0.632–0.841], 0.799 [95%-CI 0.704–0.893] and 0.707 [95%-CI 0.597–0.817], respectively (Fig. 2a). For all markers, the highest likelihood ratio was observed at 98.0% specificity. Using this fixed specificity of 98.0%, *SNAP91* had the highest sensitivity (46.5% [95%-CI 31.2–62.4%], cutoff = 112.6 copies/µl) followed by *GDNF* (sensitivity: 41.9% [95%-CI 27.0–57.9%], cutoff = 21.16 copies/µl) and *SORCS1* (sensitivity: 41.9% [95%-CI 27.0–57.9%], cutoff = 110.0 copies/µl), *SLC35F3* (sensitivity: 39.5% [95%-CI 25.0–55.6%], cutoff = 63.82 copies/µl) and *HAND2* (sensitivity: 32.6% [95%-CI 19.1–48.5%], cutoff = 79.02 copies/µl) (Fig. 2b). The association between the methylation status and clinicopathological features is shown in Table 2.

**Table 2:** Promoter methylation markers in colorectal carcinomas compared with clinicopathological features

	*GDNF*		*HAND2*		*SLC35F3*		*SNAP91*		*SORCS1*	
	M	U	M	U	M	U	M	U	M	U
	*n* (%)	*n* (%)	*n* (%)	*n* (%)	*n* (%)	*n* (%)	*n* (%)	*n* (%)	*n* (%)	*n* (%)
Tumors										
Number	17 (40.5)	25 (59.5)	11 (26.2)	31 (73.8)	15 (35.7)	27 (64.3)	19 (45.2)	23 (54.8)	17 (40.5)	25 (59.5)
Sex										
Male	11 (64.7)	17 (68.0)	8 (72.7)	20 (64.5)	10 (66.7)	18 (66.7)	11 (57.9)	17 (73.9)	10 (58.9)	18 (72.0)
Female	6 (35.3)	8 (32.0)	3 (27.3)	11 (35.5)	5 (33.3)	9 (33.3)	8 (42.1)	6 (26.1)	7 (41.1)	7 (28.0)
*P* value	0.824	0.620	1.000	0.273	0.374					
Cancer stage										
Stage I	0 (0.0)	0 (0.0)	0 (0.0)	0 (0.0)	0 (0.0)	0 (0.0)	0 (0.0)	0 (0.0)	0 (0.0)	0 (0.0)
Stage II	5 (31.3)	2 (8.0)	2 (20.0)	5 (16.1)	3 (21.4)	4 (14.8)	6 (33.3)	1 (4.3)	4 (25.0)	3 (12.0)
Stage III	8 (50.0)	16 (64.0)	5 (50.0)	19 (61.3)	7 (50.0)	17 (63.0)	8 (44.5)	16 (69.6)	8 (50.0)	16 (64.0)
Stage IV	3 (18.7)	7 (28.0)	3 (30.0)	7 (22.6)	4 (28.6)	6 (22.2)	4 (22.2)	6 (26.1)	4 (25.0)	6 (24.0)
P value	0.153	0.818	0.721	**0.047**	0.523					
Tumor location										
Proximal	0 (0.0)	14 (60.9)	1 (9.1)	13 (46.4)	0 (0.0)	14 (56.0)	0 (0.0)	14 (66.7)	0 (0.0)	14 (60.9)
Distal	16 (100.0)	9 (39.1)	10 (90.9)	15 (53.6)	14 (100.0)	11 (44.0)	18 (100.0)	7 (33.3)	16 (100.0)	9 (39.1)
*P* value	0.000	0.029	0.000	0.000	0.000					
Age (years)										
Mean age (± SD)	71 (± 7.6)	70 (± 12.2)	73 (± 7.7)	70 (± 11.2)	70 (± 9.6)	71 (± 11.1)	70 (± 9.0)	71 (± 11.7)	70 (± 9.0)	71 (± 11.5)
P value	0.927	0.333	0.812	0.663	0.885					

Pearson's chi-square (sex, stage and tumor location) and independent samples *t* tests (age) were used to calculate *P* values

To determine the best diagnostic panel, all possible marker combinations were analyzed. The panel detecting *GDNF* or *SNAP91* methylation was the optimal panel with 48.8% sensitivity [95%-CI 33.9–63.7%] and 98.0% specificity (AUC 0.727 [95%-CI 0.621–0.834], Fig. 2c, d). Without predetermined cutoffs, a panel of *SLC35F3* and *SNAP91* appeared the best performing marker panel (sensitivity 65.1%, specificity 82.0%, AUC 0.827 [95%-CI 0.701–0.954], Fig. 2e, f). Other marker combinations were observed with higher sensitivities (67.4–81.4%), however, but with reduced specificities (65.3–81.6%).

### The performance of the in silico identified DNA methylation markers and NDRG4 in combination with FIT

To determine the added diagnostic value to currently applied diagnostic assays, the marker panel was combined with our previously identified and established methylation marker *NDRG4* [27] and the FIT.

**Fig. 3:**
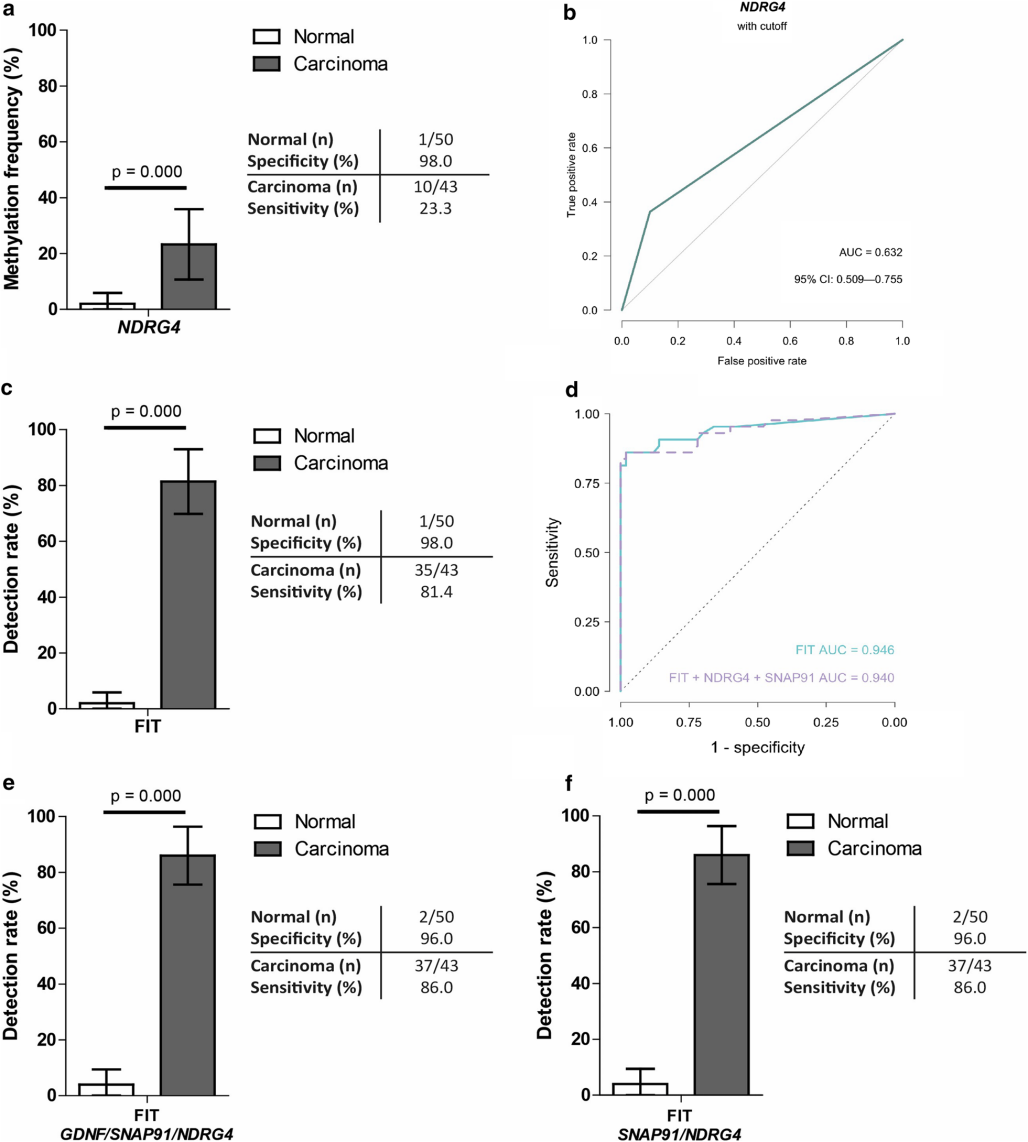
NDRG4 methylation and FIT performance in fecal DNA and combined with the previously established marker panel. **a** ROC curve for NDRG4. The jagged (green) lines indicate the ROC curve. The gray line represents the line of no discrimination between good and bad classification. **b** Methylation frequency (%) of NDRG4 methylation markers in fecal DNA of healthy controls (white bar) and carcinomas (dark gray bar). Pearson’s chi-square test was used to calculate *P* value. **c** The performance of FIT within the study population containing normal (*n* = 50) and carcinoma (*n* = 43) samples. **d** ROC curve for FIT alone and for FIT with GDNF/SNAP91/NDRG4. The green lines indicate the ROC curve for FIT alone while the purple line indicates the ROC for FIT/NDRG4/SNAP91. The gray line represents the line of no discrimination between good and bad classification*. e***, f** The best performing marker panels GDNF/SNAP91/NDRG4 (**e**) and SNAP91/NDRG4 (**f**) in combination with FIT. For all figures, the 95% CI is shown with the error bars. Pearson’s chi-square test was used to calculate *P* values

Adding ***NDRG4*** methylation (Fig. 3a, b) to the panel leads to a slightly higher AUC (0.745; [95%-CI 0.624–0.867]) and sensitivity (51.2% [95%-CI 36.3–66.1%]) without changes in specificity (98.0%), as compared to *GDNF* and *SNAP91* methylation alone (Additional file 3: Figure S2a, S2b). No other panel outperformed the *GDNF/SNAP91/NDRG4* panel.

Using a cutoff of 50 ng Hb/ml buffer, **FIT** sensitivity for CRC detection was higher than any of the described single markers or marker panels alone; sensitivity 81.4% [95%-CI 69.8–93.0%] at a specificity of 98.0%, AUC 0.946 (Fig. 3c, d). Combinations of single markers and FIT showed higher sensitivities (86.0% [95%-CI 75.7–96.4%] for *GDNF*, *SNAP91* and *SORCS1*, and 83.7% [95%-CI 72.7–94.8%] for *SLC35F3* but with a lower specificity (96.0%) (Additional file 3: Figure S2c).

When combining the best performing marker panel (*GDNF/SNAP91/NDRG4*) with FIT, the AUC reached 0.940 and the sensitivity increased to 86.0% [95%-CI 75.6–96.4%] at 96.0% specificity (Fig. 3d, e), which is higher compared to the marker panel alone (37% increase) and slightly higher than the FIT alone (4.6% increase). Interestingly, the panel combining *SNAP91*/*NDRG4*/FIT achieved the same performance (Fig. 3f) with no other panels outperforming this combination (Additional file 1: Table S2).

### Gene ontology enrichment and pathway analysis of DNA methylation markers for CRC

When investigating gene functions, we observed that twelve out of the top twenty (60.0%) genes were related to the nervous system based on their gene ontologies (Additional file 1: Table S3), resembling the previously identified neuronal-specific expression of *NDRG4* [27–29].

**Fig. 4:**
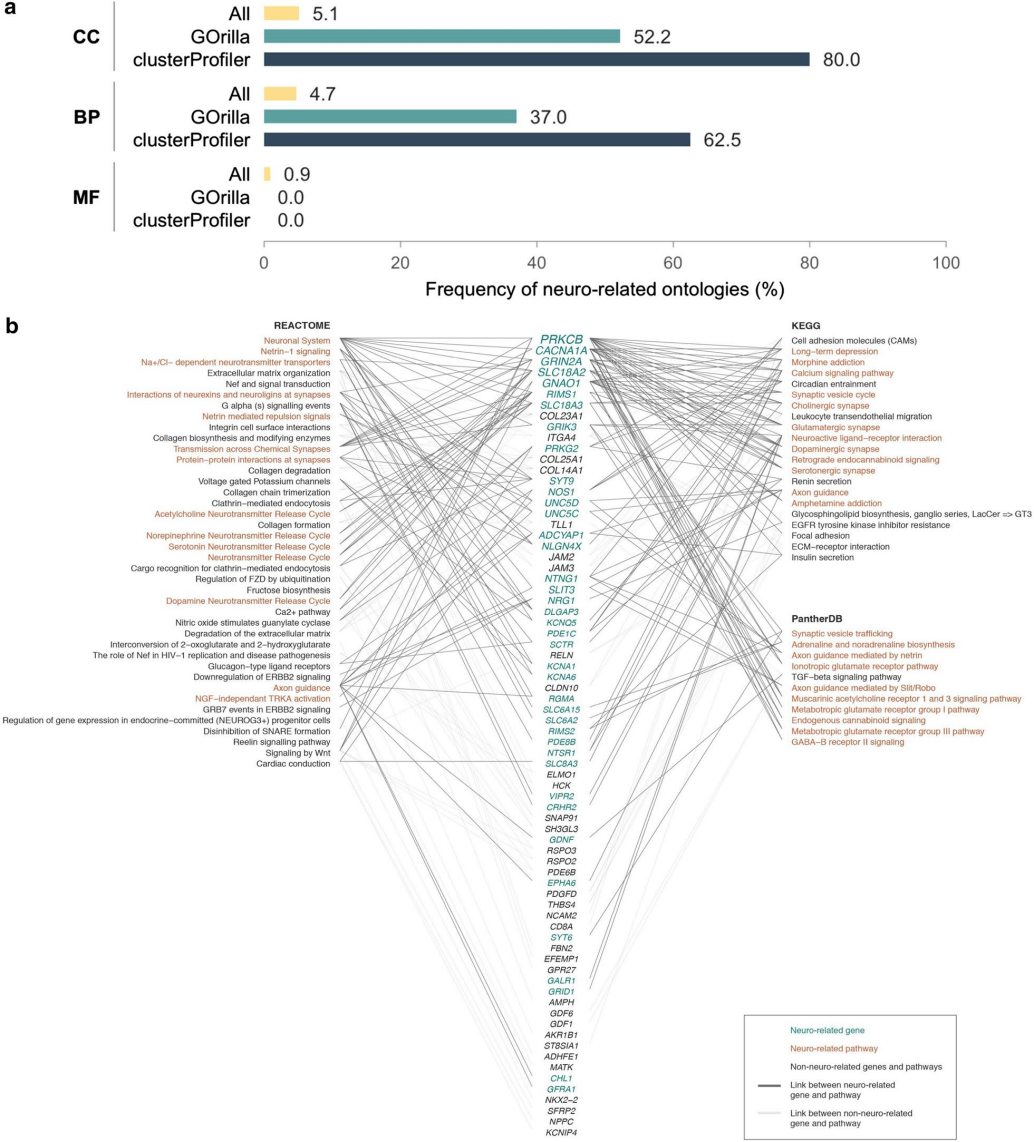
Gene ontology enrichment and pathway analysis of the full set (*n* = 221) of identified methylation markers. **a** Comparisons of the frequency of neuro-related gene ontologies in the complete set of gene ontologies (yellow bar) versus the enriched sets obtained via analysis by GOrilla (green) and clusterProfiler (dark blue). The three major subsets of gene ontologies have been accounted for: cellular component (CC), biological process (BP) and molecular function (MF). **b** Pathway analysis was performed using the ToppGene tool based on 221 genes with potential as early detection biomarkers. ToppGene links, gene lists with pathways described in three major pathway databases (KEGG pathway, Reactome and PantherDB). Nervous system-related genes are highlighted in green, nervous system-related pathways in orange and genes and pathways with a neuronal background are linked with black lines, other with gray lines

Gene ontology enrichment analyses were performed to investigate whether neuronal-related gene ontologies were also enriched in the 221 genes (Fig. 1). Using GOrilla, 46 biological process ontologies were found to be enriched, including seventeen neuronal-related ontologies (37.0%). Using clusterProfiler, 20/32 (62.5%) of the enriched ontologies were neuronal-related. Additionally, multiple neuronal-related cellular component ontologies were found to be enriched: 12/23 (52.2%) and 8/10 (80.0%), respectively. No neuronal-related molecular function ontologies were enriched in our target gene set. To put these numbers into context, we compared the frequency of neuronal gene ontologies between our lists of enriched gene ontologies and the complete set of all 47,133 ontologies. For the biological process ontologies, 4.7% of all ontologies were nervous system related in the complete set compared to 37.0% (GOrilla) and 62.5% (clusterProfiler) in the enriched set (Fig. 4a). Similar to the biological process ontologies, the frequency of neuronal-related cellular component ontologies was increased in the enriched set (52.2–80.0%) as only 5.5% of all ontologies were neuronal-related in the complete set (Fig. 4a).

Using the ToppFun tool (ToppGene Suite) and three major pathway databases (Reactome, KEGG and PantherDB), pathway analyses were performed on the 221 genes confirming that neuronal-related pathways are highly prominent. Analysis of the Reactome database revealed 14/40 neuronal-related pathways (35.0%). Higher percentage of nervous system-related pathways were observed in the KEGG (12/21 (57.1%)) and PantherDB (10/11 (90.9%)) pathway databases. More specifically, analysis using the Reactome database revealed pathways involved in synaptic protein interactions related to the following genes: *SYT9, DLGAP3, NLGN4X, GRIN2A.* Both the Reactome and KEGG databases identified pathways linked to neurotransmitter release with genes like *RIMS1, SLC18A2* and *SLC18A3* (Fig. 4b). Similarly, the PantherDB and the KEGG pathway databases analyses primarily identified pathways involved in neurotransmitter (receptor) signaling, particularly involving muscarinic acetylcholine, glutamate, GABA, serotonin and dopamine signaling (*PRKCB*, *GNAO1*, *CACNA1A*). Additionally, genes involved in synaptic vesicle trafficking were identified such as: *RIMS1* and its family member *RIMS2* next to *SYT6*, a family member of the aforementioned *SYT9*.

## Discussion

Here, we used the publicly available TCGA data to identify novel DNA methylation markers for early, noninvasive detection of CRC [8]. All five identified methylation markers (*GDNF, HAND2, SLC35F3, SNAP91* and *SORCS1)* were highly methylated in CRC samples but not in normal tissue. *GDNF*, *HAND2*, *SNAP91* and *SORCS1* have already been reported to be methylated in CRC tissue compared to normal tissue [30–33]. In stool samples from CRC patients, sensitivities decreased, ranging from 32.6% (*HAND2*) to 47.7% (*SNAP91*) at 98.0% specificity. This might be caused by the large amounts of bacterial DNA, multiple PCR-inhibiting substances such as polysaccharides, cell debris, proteins and bile salts in stool [34, 35], and the small amounts of human DNA. The sensitivity could possibly be improved by using more sensitive methods for DNA methylation detection, such as the Discrimination of Rare EpiAlleles by Melt (DREAMing) technique [36] or digital PCR analyses [37].

Before biomarkers can be developed for use in clinical practice, comparison to the current golden standard is essential to draw conclusions on their diagnostic value [19, 20, 27, 38–40]. Combining our markers with FIT yielded a slightly improved CRC detection rate using a fixed specificity; however, this needs to be validated in larger populations. The addition of our previously identified marker, *NDRG4,* improved the performance of the stool-based panel. Future studies aiming to specifically identify DNA methylation markers complementary to *NDRG4* using the publicly available TCGA data might even further improve the panel performance. Moreover, complementing our DNA methylation biomarkers with, for example, protein [41] or genetic [42] stool biomarkers could increase diagnostic performance. Finally, it should be considered that the performance of the DNA methylation markers in this study was based on the highest likelihood ratio, corresponding to 98.0% specificity. This was associated with lower sensitivities ranging from 32.9 to 46.5%. However, within the research field of biomarker development, there is ample discussion on how the selection of cutoff values should be performed [43]. Although data-driven methods to maximize the diagnostic value of biomarkers are suggested, e.g., likelihood ratio and Youden index, decision-driven cutoff values matching a specific sensitivity or specificity are also used [43]. Using a predetermined cutoff matching a lower specificity could lead to an increase in sensitivity as the choice of cutoff is always a trade-off between sensitivity and specificity [43].

Among the in silico identified, potential diagnostic DNA methylation biomarkers, we found a significant enrichment of nervous system-related pathways and gene ontologies. This is in accordance with previous data [31, 44]. Note that gene ontologies are structured as a hierarchical tree, meaning that different ontologies often share a significant number of genes and that they are therefore not independent. This impedes a statistical interpretation of the differences in the frequencies of neuro-related ontologies. Nevertheless, these results paint an intriguing picture of the potential role of the nervous system in CRC. Interestingly, all five of the markers assessed in this study are related to the nervous system, either by expression location, biological role or association with neurodegenerative disease. Previously, we observed that *NDRG4,* one of the Cologuard® markers [27], is specifically expressed in nervous systems [28]. Although the role of the nervous system in CRC is understudied, several landmark papers have shown the importance of nerves in different types of cancer, promoting cancer growth, migration and invasion [32, 45–49]]. However, further studies are necessary to elucidate why the promoter regions of these neuronal genes are frequently methylated in CRC and whether this has biological consequences. Finally, further validation of our identified genes as CRC biomarkers is needed to investigate their added value to current screening tests. Moreover, their potential to detect early stage lesions, e.g., advanced adenomas, is yet to be studied.

## Supplementary Information

**Additional file 1**. Supplementary methods and tables.

**Additional file 2**. Early detection methylation marker validation using carcinoma and matched normal tissue from CRC patients.

**Additional file 3**. FIT and NDRG4 methylation performance in fecal DNA in combination with either the single markers or established marker panel.

## Abbreviations

AUC: Area under the curve
BMP3: Bone morphogenetic protein 3
BRCA: Breast cancer
CI: Confidence interval
CRC: Colorectal cancer
DREAMing: Discrimination of Rare EpiAlleles by Melt
FDR: False-discovery rate
FFPE: Formalin-fixed paraffin embedded
FIT: Fecal immunochemical test
GDNF: Glial-derived neurotrophic factor
HAND2: Heart and neural crest derived 2
MSP: Methylation-specific PCR
NDRG4: N-myc downstream-regulated gene 4
PCR: Polymerase chain reaction
ROC: Receiver operating characteristics
SLC35F3: Solute carrier family 35 member F3
SNAP91: Synaptosome-associated protein 91
SORCS1: Sortilin-related VPS10 domain containing receptor 1
TCGA: The Cancer Genome Atlas
