# Supplementary material for: Identification of DNA methylation markers for early detection of CRC indicates a role for nervous system-related genes in CRC
Source: Clin Epigenetics. 2021 Apr 15;13:80. doi: 10.1186/s13148-021-01067-9 (PMC8048074; doi:10.1186/s13148-021-01067-9)
Supplement: Supplementary file 1 — Additional file 1. Supplementary methods and tables. [file 13148_2021_1067_MOESM1_ESM.docx]

**Supplementary Data**

**Identification of DNA methylation markers for early detection of CRC indicate a role for nervous system-related genes in CRC**

**Neuronal-related genes identified as DNA methylation CRC markers**

Glenn Rademakers^1*^, Maartje Massen^1*^, Alexander Koch^1*^, Muriel X. Draht^1^, Nikkie Buekers^1^, Kim A.D. Wouters^1^, Nathalie Vaes^1^, Tim De Meyer^2^, Beatriz Carvalho^3^, Gerrit A. Meijer^3^, James G. Herman^4^, Kim M. Smits^1^, Manon van Engeland^1^, Veerle Melotte^1,5^

*Affiliations of authors:*

*^1^Department of Pathology, GROW – School for Oncology and Developmental Biology, Maastricht University Medical Center, Maastricht; The Netherlands*

*^2^ Department of Data Analysis & Mathematical Modelling, Ghent University, Ghent, Belgium.*

*^3^ Department of Pathology, Netherlands Cancer Institute, Amsterdam, the Netherlands.*

*^4^ University of Pittsburgh Cancer Institute, The Hillman Cancer Center, Pittsburgh, PA, USA*

*^5^ Department of Clinical Genetics; Erasmus University Medical Center, Rotterdam; The Netherlands*

* Authors contributed equally

* Authors contributed equally

**Supplementary methods**

**DNA methylation marker validation**

*DNA isolation of tissue samples*

A 5µm formalin-fixed paraffin-embedded (FFPE) tissue section was stained with hematoxylin and eosin and revised by an experienced gastro-intestinal pathologist. Five 20µm thick sections were deparaffinized and macro dissected for genomic DNA extraction with the QIAamp mini kit (51304; Qiagen) according to the manufacturer’s protocol.

*Collection and DNA isolation of stool samples*

Within 72h after collection, fecal samples were processed to achieve a final stool:buffer w/v ratio of 1:7. Samples were then split in aliquots and stored at -80°C. For DNA isolation, the stool DNA was first precipitated, then isolated with the QIAamp Fast DNA Stool Mini kit (51604; Qiagen) making use of the QIAcube (Qiagen), according to the manufacturer’s protocol. Briefly, two ml of homogenized stool were pre-cleaned using a precipitation protocol in order to minimize solid material and bacterial DNA. Next, samples were centrifuged for 10 minutes at 14,000 rpm. The supernatant was collected and 200µl 3M sodium acetate (pH = 5.2) and 2000µl 100% isopropanol was added. A cell/DNA pellet was collected at 14,000 rpm for 5 minutes, followed by a wash step with 70% ethanol (1ml). The precipitated pellet was dissolved in 200µl 1x TE buffer and concentrations were measured by NanoDrop 2000 spectrophotometer (Thermofisher Scientific) after which the samples were stored at -80°C.

*Sodium bisulfite conversion & (quantitative) methylation-specific PCR ((q)MSP)*

Sodium bisulfite conversion was carried out on 500ng genomic DNA isolated from tissue samples or 2µg DNA isolated from stool samples using the Qiagen EpiTect Bisulfite kit (59104; Qiagen) on the QIAcube. Following sodium bisulfite conversion, nested methylation-specific PCRs (MSPs) were carried out on 100ng bisulfite converted tissue DNA as described by Herman et al. (1996) and Van Engeland et al. (2003).

For the qMSP, normalization of the qMSP data was performed using Alu repeat elements as reference. Each reaction was carried out in a final volume of 25µl, consisting of 2.0µl bisulfite converted DNA, 17.05µl sterile H2O, 2.5µl 10x Magic Buffer, 1.0 µl dNTPs (6.25mM; BIO-39026; Bioline Meridian Bioscience), 0.75 µl forward primer (10µM), 0.75 µl reverse primer (10µM), 0.5 µl probe (5µM) and 0.2 µl HS Taq (5U/µl; BIO-21112; Bioline Meridian Bioscience). In case of stool samples, 0.25 µl Spermidine (05292-1ML-F; Sigma-Aldrich/Merck) was added to facilitate the PCR reaction *(Kikuchi et al., Biochemical genetics, 2010; Roperch et al., BMC Biotechnology, 2015)*. The PCR program was initiated with 3 minutes at 95°C, followed by 50 cycles of 15 seconds at 95°C, 1 minute at 60°C and 1 minute at 72°C using the C1000 Touch Thermal Cycler (Biorad Laboratories). A serially diluted plasmid (Life Technologies) containing the target amplicons and the reference amplicon was used as a standard curve (4.00E+07 copies/μl, 4.00E+06 copies/μl, 4.00E+05 copies/μl, 4.00E+04 copies/μl, 4.00E+03 copies/μl, 4.00E+02 copies/μl, 4.00E+01 copies/μl). Quantification of DNA methylation was analyzed in a blinded manner by interpolating the Ct value of the unknown sample to the corresponding plasmid copy using Biorad CFX manager 2.0 software (Biorad Laboratories). For both MSP and qMSP analysis, primer pairs were designed, using both Primer3 and Gene Runner, in close proximity to the TCGA-derived differentially methylated Infinium 450K probes, and were manufactured by Eurogentec (supplementary table 1 (MSP) and 2 (qMSP)).

*Methylation data and statistical analyses*

The likelihood ratio equals sensitivity/(100%-specificity) and estimates the probability of correctly predicting disease in ratio to the probability of incorrectly predicting disease. Through the likelihood ratio the corresponding sensitivity and specificity could be derived.

Furthermore, to assess the association between promoter methylation of the selected genes in CRC tissue and clinicopathological features, the Pearson chi-square test for categorical variables (sex, cancer stage, tumor location) and independent samples t-test for continuous variables (age) were used.

**Additional Files 2-3 - Figures Legends**

**Figure S1:** **Early detection methylation marker validation using carcinoma and matched normal tissue from CRC patients.** Methylation frequency (%) for all five individual methylation markers (*GDNF, HAND2, SLC35F3, SNAP91 and SORCS1*) in normal tissue (white bars) and carcinoma tissue (black bars) from CRC patients. For both normal and carcinoma tissue, the number of methylated samples are indicated in the table together with the methylation frequency (%) for each tested marker. McNemar’s test was used to calculate P-values.

**Figure S2:** **FIT and *NDRG4* methylation performance in fecal DNA in combination with either the single markers or established marker panel.** A. ROC curve for GDNF/SNAP91/NDRG4 marker panel. The jagged (green) lines indicate the ROC curve. The grey line represents the line of no discrimination between good and bad classification. B. Methylation frequency (%) of GDNF/SNAP91/NDRG4 methylation marker panel in fecal DNA healthy controls (white bar) and carcinomas (dark grey bar). Fisher’s Exact test was used to calculate P-value. C. The detection rate of the FIT in combination with either GDNF, HAND2, SLC35F3, SNAP91, SORCS1 or NDRG4 in both normal samples and carcinoma samples.

**Table S1:** **Clinicopathological features of the (matched normal and CRC) tissue samples obtained from a hospital-based series****.*

| **Patient Demographics** | **Carcinoma (n = 34) (matched with normals)**  **n (%)** |  |
| --- | --- | --- |
| **Sex** |  |  |
| Male | 18 (52.9) |  |
| Female | 16 (47.1) |  |
| **Age (years)** |  |  |
| Mean age (SD) | 70.0 (±9.2) |  |
| **Cancer stage** |  |  |
| Stage I | 6 (17.6%) |  |
| Stage II | 13 (38.2%) |  |
| Stage III | 9 (26.5%) |  |
| Stage IV | 6 (17.6%) |  |
| **Location** |  |  |
| Colon ascendens | 14 (41.2%) |  |
| Colon transversum | 4 (11.8%) |  |
| Sigmoid | 10 (29.4%) |  |
| Rectosigmoid | 4 (11.8%) |  |
| Rectum | 2 (5.9%) |  |
| **Differentiation grade** |  |  |
| Poor | 2 (5.9%) |  |
| Moderate | 28 (82.4%) |  |
| Well | 4 (11.8%) |  |
| **Histology** |  |  |
| Adenocarcinoma | 26 (76.5%) |  |
| Mucinous adenocarcinoma | 8 (23.5%) |  |
| * Tissue retrieved retrospectively from the tissue archive of the department of  Pathology of the Maastricht University Medical Center | | |

**Table S2:** All possible combinations of the identified DNA methylation markers with FIT

| **FIT Performance** | |  |  |  |  |  |  |  |  |  |  |  |  |  |  |  |  |  |  |  |
| --- | --- | --- | --- | --- | --- | --- | --- | --- | --- | --- | --- | --- | --- | --- | --- | --- | --- | --- | --- | --- |
|  | **FIT** |  |  |  |  |  |  |  |  |  |  |  |  |  |  |  |  |  |  |  |
| **Specificity** | 98,0 |  |  |  |  |  |  |  |  |  |  |  |  |  |  |  |  |  |  |  |
| **Sensitivity** | 81,4 |  |  |  |  |  |  |  |  |  |  |  |  |  |  |  |  |  |  |  |
|  |  |  |  |  |  |  |  |  |  |  |  |  |  |  |  |  |  |  |  |  |
| **Methylation marker performance** | | | | | | |  |  |  |  |  |  |  |  |  |  |  |  |  |  |
|  | **GDNF** | **HAND2** | **SLC35F3** | **SNAP91** | **SORCS1** | **NDRG4** |  |  |  |  |  |  |  |  |  |  |  |  |  |  |
| **Specificity** | 98,0 | 98,0 | 98,0 | 98,0 | 98,0 | 98,0 |  |  |  |  |  |  |  |  |  |  |  |  |  |  |
| **Sensitivity** | 41,9 | 32,6 | 39,5 | 46,5 | 41,9 | 37,2 |  |  |  |  |  |  |  |  |  |  |  |  |  |  |
|  |  |  |  |  |  |  |  |  |  |  |  |  |  |  |  |  |  |  |  |  |
| **FIT - One methylation marker** | | | | | | |  |  |  |  |  |  |  |  |  |  |  |  |  |  |
|  | **FIT GDNF** | **FIT HAND2** | **FIT SLC35F3** | **FIT SNAP91** | **FIT SORCS1** | **FIT NDRG4** |  |  |  |  |  |  |  |  |  |  |  |  |  |  |
| **Specificity** | 96,0 | 96,0 | 96,0 | 96,0 | 96,0 | 96,0 |  |  |  |  |  |  |  |  |  |  |  |  |  |  |
| **Sensitivity** | 86,0 | 81,4 | 83,7 | 86,0 | 86,0 | 86,0 |  |  |  |  |  |  |  |  |  |  |  |  |  |  |
|  |  |  |  |  |  |  |  |  |  |  |  |  |  |  |  |  |  |  |  |  |
| **FIT - Two methylation markers** | | | | | | | | | | | | | | | |  |  |  |  |  |
|  | **FIT GDNF HAND2** | **FIT GDNF SLC35F3** | **FIT GDNF SNAP91** | **FIT GDNF SORCS1** | **FIT GDNF NDRG4** | **FIT  HAND2 SLC35F3** | **FIT HAND2 SNAP91** | **FIT HAND2 SORCS1** | **FIT HAND2 NDRG4** | **FIT  SLC35F3 SNAP91** | **FIT SLC35F3 SORCS1** | **FIT SLC35F3 NDRG4** | **FIT SNAP91 SORCS1** | **FIT SNAP91 NDRG4** | **FIT SORCS1 NDRG4** |  |  |  |  |  |
| **Specificity** | 94,0 | 96,0 | 96,0 | 96,0 | 96,0 | 94,0 | 94,0 | 94,0 | 94,0 | 96,0 | 96,0 | 96,0 | 96,0 | 96,0 | 96,0 |  |  |  |  |  |
| **Sensitivity** | 86,0 | 86,0 | 86,0 | 86,0 | 86,0 | 83,7 | 86,0 | 86,0 | 86,0 | 86,0 | 86,0 | 86,0 | 86,0 | 86,0 | 86,0 |  |  |  |  |  |

|  |  |  |  |  |  |  |  |  |  |  |  |  |  |  |  |  |  |  |  |  |
| --- | --- | --- | --- | --- | --- | --- | --- | --- | --- | --- | --- | --- | --- | --- | --- | --- | --- | --- | --- | --- |
| **FIT - Three methylation markers** | | | | | | | | | | | | | | | | | | | | |
|  | **FIT GDNF HAND2 SLC35F3** | **FIT GDNF HAND2 SNAP91** | **FIT GDNF HAND2 SORCS1** | **FIT GDNF HAND2 NDRG4** | **FIT GDNF SLC35F3 SNAP91** | **FIT GDNF SLC35F3 SORCS1** | **FIT GDNF SLC35F3 NDRG4** | **FIT GDNF SNAP91 SORCS1** | **FIT GDNF SNAP91 NDRG4** | **FIT GDNF SORCS1 NDRG4** | **FIT HAND2 SLC35F3 SNAP91** | **FIT HAND2 SLC35F3 SORCS1** | **FIT HAND2 SLC35F3 NDRG4** | **FIT HAND2 SNAP91 SORCS1** | **FIT HAND2 SNAP91 NDRG4** | **FIT HAND2 SORCS1 NDRG4** | **FIT SLC35F3 SNAP91 SORCS1** | **FIT SLC35F3 SNAP91 NDRG4** | **FIT SLC35F3 SORCS1 NDRG4** | **FIT SNAP91 SORCS1 NDRG4** |
| **Specificity** | 94,0 | 94,0 | 94,0 | 94,0 | 96,0 | 96,0 | 96,0 | 96,0 | 96,0 | 96,0 | 94,0 | 94,0 | 94,0 | 94,0 | 94,0 | 94,0 | 96,0 | 96,0 | 96,0 | 96,0 |
| **Sensitivity** | 86,0 | 86,0 | 86,0 | 86,0 | 86,0 | 86,0 | 86,0 | 86,0 | 86,0 | 86,0 | 86,0 | 86,0 | 86,0 | 86,0 | 86,0 | 86,0 | 86,0 | 86,0 | 86,0 | 86,0 |

| **FIT - Four methylation markers** | | | | | | | | | | | | | | |
| --- | --- | --- | --- | --- | --- | --- | --- | --- | --- | --- | --- | --- | --- | --- |
|  | **FIT GDNF HAND2 SLC35F3 SNAP91** | **FIT GDNF HAND2 SLC35F3 SORCS1** | **FIT GDNF HAND2 SLC35F3 NDRG4** | **FIT GDNF HAND2 SNAP91 SORCS1** | **FIT GDNF HAND2 SNAP91 NDRG4** | **FIT GDNF HAND2 SORCS1 NDRG4** | **FIT GDNF SLC35F3 SNAP91 SORCS1** | **FIT GDNF SLC35F3 SNAP91 NDRG4** | **FIT GDNF SLC35F3 SORCS1 NDRG4** | **FIT GDNF SNAP91 SORCS1 NDRG4** | **FIT HAND2 SLC35F3 SNAP91 SORCS1** | **FIT HAND2 SLC35F3 SNAP91 NDRG4** | **FIT HAND2 SNAP91 SORCS1 NDRG4** | **FIT SLC35F3 SNAP91 SORCS1 NDRG4** |
| **Specificity** | 94,0 | 94,0 | 94,0 | 94,0 | 94,0 | 94,0 | 96,0 | 96,0 | 96,0 | 96,0 | 94,0 | 94,0 | 94,0 | 96,0 |
| **Sensitivity** | 86,0 | 86,0 | 86,0 | 86,0 | 86,0 | 86,0 | 86,0 | 86,0 | 86,0 | 86,0 | 86,0 | 86,0 | 86,0 | 86,0 |
|  |  |  |  |  |  |  |  |  |  |  |  |  |  |  |
| **FIT - Five methylation markers** | | | | | | |  |  |  |  |  |  |  |  |
|  | **FIT GDNF HAND2 SLC35F3 SNAP91 SORCS1** | **FIT GDNF HAND2 SLC35F3 SNAP91 NDRG4** | **FIT GDNF HAND2 SLC35F3 SORCS1 NDRG4** | **FIT GDNF HAND2 SNAP91 SORCS1 NDRG4** | **FIT GDNF SLC35F3 SNAP91 SORCS1 NDRG4** | **FIT HAND2 SLC35F3 SNAP91 SORCS1 NDRG4** |  |  |  |  |  |  |  |  |
| **Specificity** | 94,0 | 94,0 | 94,0 | 94,0 | 96,0 | 94,0 |  |  |  |  |  |  |  |  |
| **Sensitivity** | 86,0 | 86,0 | 86,0 | 86,0 | 86,0 | 86,0 |  |  |  |  |  |  |  |  |
|  |  |  |  |  |  |  |  |  |  |  |  |  |  |  |
| **FIT - All methylation markers** | |  |  |  |  |  |  |  |  |  |  |  |  |  |
|  | **FIT GDNF HAND2 SLC35F3 SNAP91 SORCS1 NDRG4** |  |  |  |  |  |  |  |  |  |  |  |  |  |
| **Specificity** | 94,0 |  |  |  |  |  |  |  |  |  |  |  |  |  |
| **Sensitivity** | 86,0 |  |  |  |  |  |  |  |  |  |  |  |  |  |

| **Gene name** | **Gene Sym.** | **Neuronal-related gene ontologies** | |
| --- | --- | --- | --- |
| Adenosine deaminase RNA specific B | *ADARB2* | - | |
| **Amphyphysin** | ***AMPH*** | **Chemical synaptic transmission** | |
| **Collagen type XXV alpha 1 chain** | ***COL25A1*** | **Axonogenesis** | |
| **Doublecortin like kinase 1** | ***DCLK1*** | **Neuron migration (Central) nervous system development Axon projection Axon extension** |  |
| **Family with sequence similarity**  **19 member A4** | ***FAM19A4*** | **Regulation of membrane potential Regulation of sensory perception of pain** | |
| Filamin binding LIM protein 1 | *FBLIM1* | - | |
| Friend leukemia integration 1 | *FLI1* | - | |
| ***GDNF*** | ***GDNF*** | **Neural crest migration Postsynaptic membrane organization (Sympathetic) nervous system development Axon guidance Peristalsis Neuron projection development Negative regulation of neuron apoptosis Enteric nervous system development Regulation dopamine uptake**  **Synaptic transmission** | |
| **Growth differentiation factor 6** | ***GDF6*** | **Positive regulation of neuron differentiation** | |
| **GDNF family receptor alpha 1** | ***GFRα1*** | **Axon guidance SNAP signaling pathway** | |
| ***HAND2*** | ***HAND2*** | **Noradrenergic neuron differentiation Sympathetic nervous system development**  **Peripheral nervous system development** | |
| **Potassium voltage-gated channel**  **subfamily A member 1** | ***KCNA1*** | **Regulation of muscle contraction Chemical synaptic transmission Neuroblast proliferation Cell communication Neuronal action potential Hippocampus development Neuronal signal transduction Neuromuscular process** | |
| **Potassium voltage-gated channel**  **subfamily Q member 5** | ***KCNQ5*** | **Chemical synaptic transmission** | |
| LON peptidase N-terminal  domain and ring finger 2 | *LONRF2* | - | |
| **Leucine rich repeat containing 7** | ***LRRC7*** | **Neuron projection development** | |
| Protein phosphatase 1 regulatory  subunit 16B | *PPP1R16B* | - | |
| *SLC35F3* | *SLC35F3* | - | |
| *SNAP91* | *SNAP91* | - | |
| ***SORCS1*** | ***SORCS1*** | **Neuropeptide signaling** | |
| Ubiquitin specific peptidase 44 | *USP44* | - | |

**Table S3:** Top 20 identified methylation markers and associated neuronal-related gene ontologies.
