# Supplementary figures and images for: Identification of DNA methylation markers for early detection of CRC indicates a role for nervous system-related genes in CRC
Source: Clin Epigenetics. 2021 Apr 15;13:80. doi: 10.1186/s13148-021-01067-9 (PMC8048074; doi:10.1186/s13148-021-01067-9)

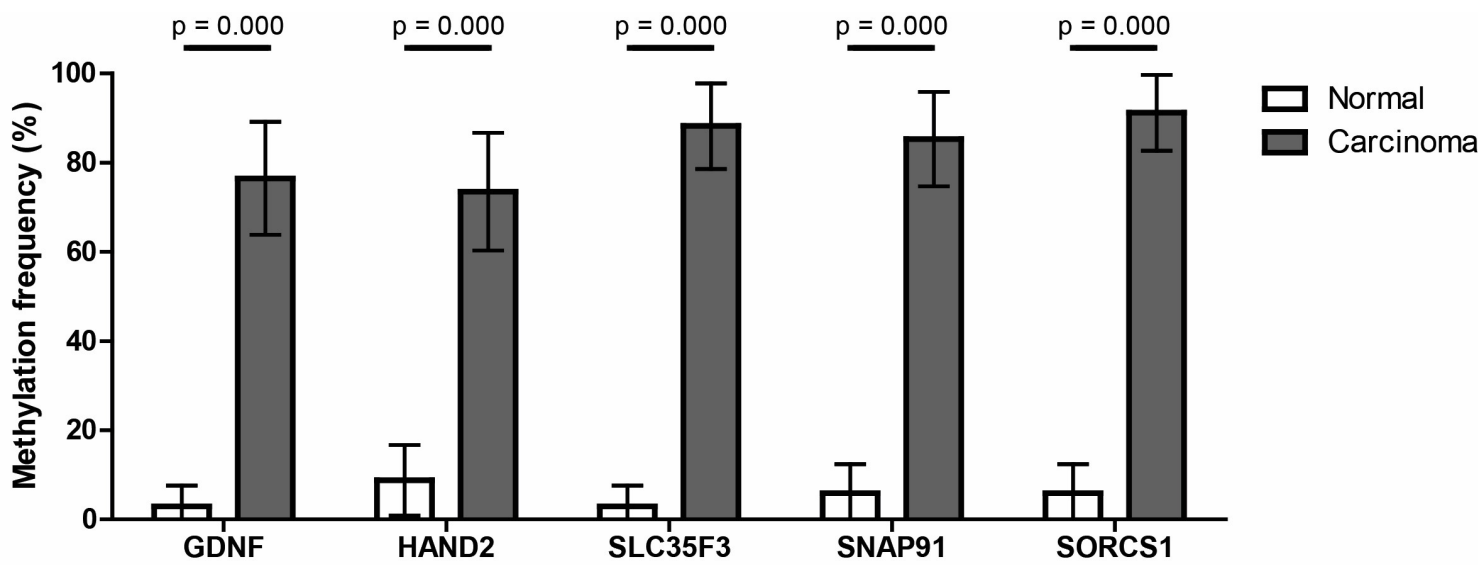

|                   |       |       |       |       |       |
|-------------------|-------|-------|-------|-------|-------|
| Normal tissue (n) | 1/34  | 3/34  | 1/34  | 2/34  | 2/34  |
| Methylated (%)    | 2.9   | 8.8   | 2.9   | 5.9   | 5.9   |
| Carcinoma (n)     | 26/34 | 25/34 | 30/34 | 29/34 | 31/34 |
| Methylated (%)    | 76.5  | 73.5  | 88.2  | 85.3  | 91.2  |

Supplement: Supplementary file 2 — Additional file 2. Early detection methylation marker validation using carcinoma and matched normal tissue from CRC patients. [file 13148_2021_1067_MOESM2_ESM.pdf]

**A**

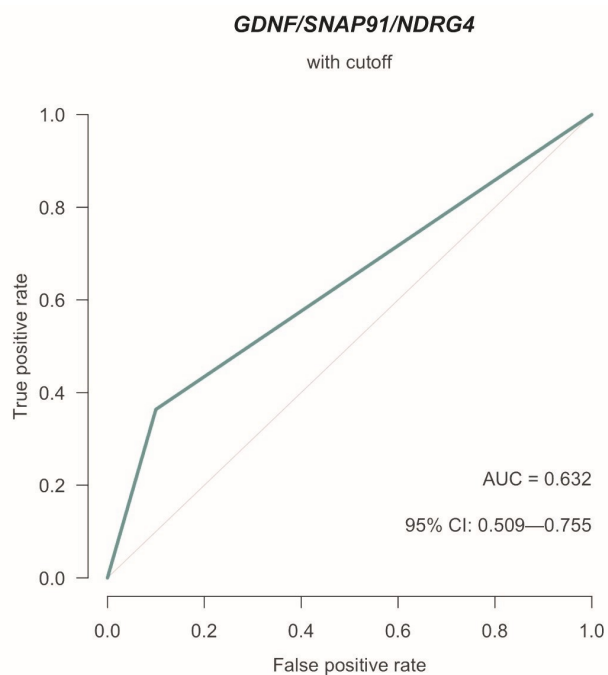

**B**

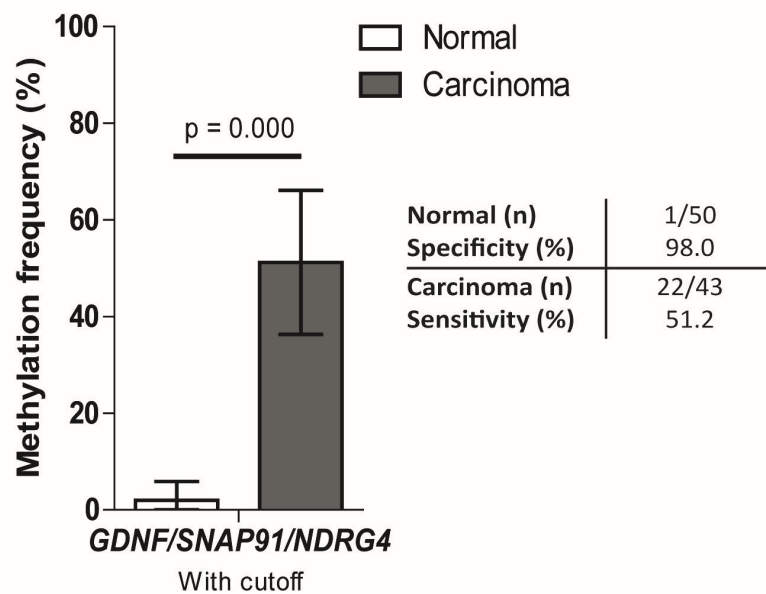

**C**

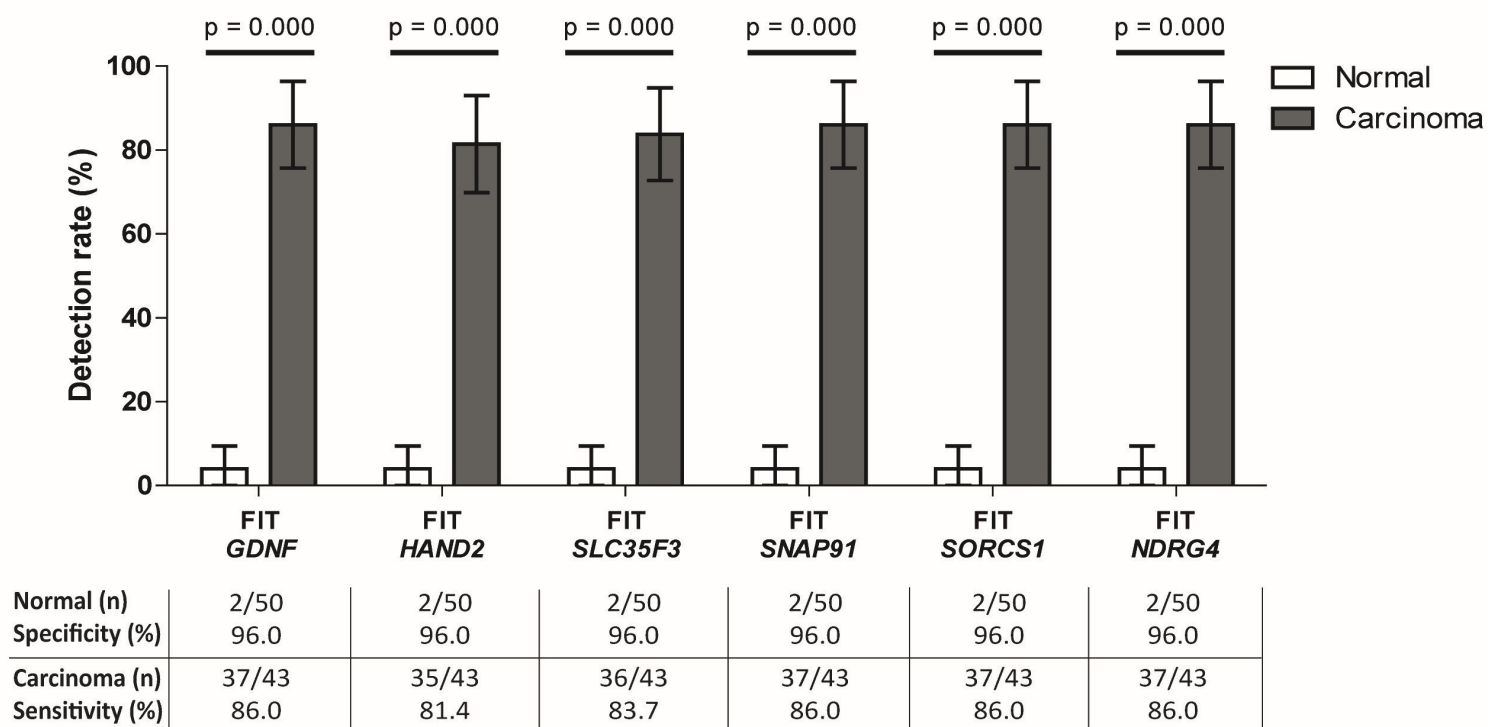

Supplement: Supplementary file 3 — Additional file 3. FIT and NDRG4 methylation performance in fecal DNA in combination with either the single markers or established marker panel. [file 13148_2021_1067_MOESM3_ESM.pdf]
